# Cross-talk between blood vessels and neural progenitors in the developing brain

**DOI:** 10.1042/NS20170139

**Published:** 2018-03-30

**Authors:** Mathew Tata, Christiana Ruhrberg

**Affiliations:** UCL Institute of Ophthalmology, 11-43 Bath Street, London EC1V 9EL, U.K.

**Keywords:** angiogenesis, blood brain barrier, central nervous system, embryogenesis, neurogenesis, neural stem cells

## Abstract

The formation of the central nervous system (CNS) involves multiple cellular and molecular interactions between neural progenitor cells (NPCs) and blood vessels to establish extensive and complex neural networks and attract a vascular supply that support their function. In this review, we discuss studies that have performed genetic manipulations of chick, fish and mouse embryos to define the spatiotemporal roles of molecules that mediate the reciprocal regulation of NPCs and blood vessels. These experiments have highlighted core functions of NPC-expressed ligands in initiating vascular growth into and within the neural tube as well as establishing the blood–brain barrier. More recent findings have also revealed indispensable roles of blood vessels in regulating NPC expansion and eventual differentiation, and specific regional differences in the effect of angiocrine signals. Accordingly, NPCs initially stimulate blood vessel growth and maturation to nourish the brain, but blood vessels subsequently also regulate NPC behaviour to promote the formation of a sufficient number and diversity of neural cells. A greater understanding of the molecular cross-talk between NPCs and blood vessels will improve our knowledge of how the vertebrate nervous system forms and likely help in the design of novel therapies aimed at regenerating neurons and neural vasculature following CNS disease or injury.

## Introduction

The formation of the central nervous system (CNS) begins early in development following the specification of the ectodermal germ layer [1]. The neural ectoderm develops into the neuroepithelium, which is initially comprised of a small pool of highly proliferative neural progenitor cells (NPCs) that will ultimately give rise to all glia and neurons in the adult nervous system via several NPC-derived lineages [2]. These NPC lineages generate functionally specialised neurons in a process termed neurogenesis, which is tightly regulated by both cell-intrinsic mechanisms and extrinsic signals from surrounding neural and non-neural cells. The neuroepithelium must also attract blood vessels to sustain the high metabolic demands of NPCs and their neuronal progeny. Like elsewhere in the body, vessel formation involves the proliferation and migration of endothelial cells (ECs) that form the inner layer of all blood vessels to contain the vascular lumen and the recruitment of supporting mural cells that are termed pericytes.

Here, we will review signals produced by the neuroepithelium that direct the growth and maturation of blood vessels in the CNS, as well as reciprocal roles of CNS vasculature in regulating NPC behaviour. We will particularly focus on studies that have explored relevant molecular mechanisms through the analysis of conditional mouse mutants, because this allows us to distinguish the relative contribution of specific factors in endothelial versus neural cells during neurogenesis. We will further highlight gaps in our current knowledge about the molecular interplay between both developing systems and provide an outlook on potential directions for future research in this field.

## Role of NPCs in brain vascularisation and vascular maturation

To ensure an adequate supply of oxygen and nutrients, the neuroepithelium has to produce chemoattractive guidance cues that ensure the formation of patent blood vessels in positions where neurons form and function.

### Key steps in brain vascularisation

**Figure 1:**
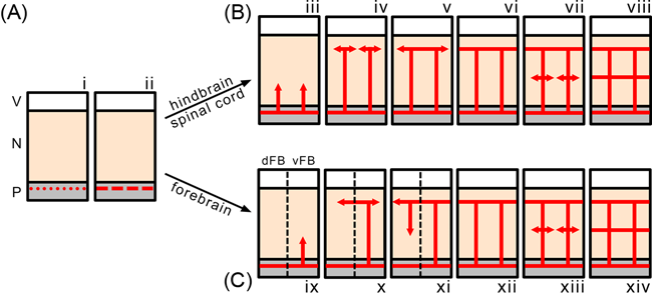
Spatial progression of mammalian CNS vascularisation (**A**) Angioblasts (red dots, i) converge and align outside the neural tube to form the PNP (red dashes, ii). (**B**) Radial vessels sprout from the PNP into hindbrain and spinal cord and grow radially towards the ventricular zone (iii). Before reaching the ventricular surface, the sprouts turn laterally (iv) and fuse into the SVP (v). After SVP formation is complete (vi), a deeper plexus sprouts from the radial vessels (vii, viii). (**C**) Vascularisation proceeds in a similar fashion in the ventral portion of the forebrain to more caudal regions of the neural tube (as shown in iii–viii), but the dorsal forebrain is vascularised by lateral SVP sprouts (x) that extend radial vessels back to the underlying PNP (xi). Abbreviations: dFB, dorsal forebrain; N, neuroepithelium; P, pial tissue; V, ventricle; vFB, ventral forebrain.

The main steps in neural tube vascularisation are by now well characterised, with many similarities and some differences along the rostrocaudal axis [3]. To vascularise the neuroepithelium, vessel sprouts ingress from a perineural vascular plexus (PNP) directly outside the pial surface of the neural tissue [4–6]. The PNP forms via vasculogenesis, a process in which angioblasts differentiate from the presomitic mesoderm to form blood vessels *de novo* [7,8] (Figure 1). Angioblasts are initially attracted to the basal surface of the neural tube by neural VEGF-A [8] (see below). The next steps of neural tube vascularisation are particularly well characterised for the hindbrain [9] and the spinal cord [10,11]. In both parts of the neural tube, vessels sprout from the PNP into the neural parenchyma, grow radially towards the ventricular zone and turn laterally before reaching the ventricular surface; the laterally sprouting vessels then fuse with one another into a periventricular vessel network known as the subventricular vascular plexus (SVP) [4–6] (Figure 1B). Subsequently, lateral sprouts emerge from radial vessels in deeper brain layers and then fuse into additional plexi. This process appears to be conserved in the zebrafish and avian hindbrain, possibly reflecting the physiological need for evolutionary conservation of hindbrain anatomy across the vertebrate phylum [12]. Studies of spinal cord vascularisation have further shown that radial vessels initially invade the neural parenchyma at several stereotypical entry points around the circumference of the spinal cord, except at the level of the ventral motor neuron (MN) columns, which remain avascular until SVP formation is complete in the remainder of the CNS [13].

Vascularisation of the forebrain differs from that of the hindbrain and spinal cord with respect to the spatiotemporal progression of vascular outgrowth [14] (Figure 1C). Radial vessels ingress initially in the ventral compartment of the telencephalic vesicle similar to the process of hindbrain vascularisation; however, even though an SVP forms in the ventrolateral forebrain, dorsomedial regions remain temporarily avascular [14]. Indeed, vascularisation of the dorsal forebrain is not initiated by vessels originating from the PNP, but via vascular spouting from the SVP in the ventral forebrain, whereby sprouts extend tangentially around the telencephalon to vascularise the dorsal regions [14].

During neural tube vascularisation at all rostrocaudal levels, ECs recruit mural cells called pericytes, which ensheath the blood vessel endothelium, but are separated from it by a basement membrane. Loss of pericytes causes excessive endothelial proliferation, but also increases endothelial apoptosis or transcytosis [26]. Accordingly, several forms of paediatric brain haemorrhage are thought to result from poor pericyte recruitment to the vascular network of the germinal matrix in human foetuses [27].

Subsequent to its formation as pericyte-ensheathed endothelial networks, the CNS vasculature undergoes specialisation. Thus, ECs extend their interaction with pericytes to astrocytes, whose endfeet make direct contact with the endothelium [28,29]. The reciprocal interaction of these three cell types results in the formation of the blood–brain barrier (BBB), which maintains essential tissue homoeostasis by establishing selective permeability to fluid, molecules and cells between the blood stream and neural parenchyma. The level of extracellular fluid in neural tissue is tightly regulated by the BBB to maintain physiological levels of water and prevent fluid build-up in the brain [30]. Glucose is actively pumped into the CNS by BBB ECs through the glucose transporter GLUT1, whose expression is regulated in part by NPC signals [18] in a mechanism that likely supports the metabolic demand of neural activity (see below). Additional transmembrane pumps are responsible for the movement of metabolites, toxins and drugs bidirectionally across the barrier [31]. The BBB also acts as a physical barrier to harmful pathogens and ensures that immune cells can only access the brain in certain physiological states to reduce the opportunity for auto-immunity [32].

### Molecules regulating brain vascular development

The processes by which new blood vessels sprout from pre-existing ones, migrate into the neural parenchyma, branch and fuse into a network and then undergo maturation is collectively termed ‘angiogenesis’ and involves regulation by specific gene families. We will highlight several of these genes below, with specific consideration given to pro-vascular molecules expressed by NPC populations (Table 1).

**Table 1:** NPC regulation of CNS vascularisation

Process	NPC signal/molecule	Species/CNS region	References
(1) Vascular ingression and intraneural branching	VEGF-A	Fish spinal cord	[15]
		Mouse forebrain	[16,17]
		Mouse mid-/hindbrain	[17]
	WNT7A/7B	Mouse spinal cord	[18,19]
(2) Branching/angiogenesis	VEGF-A	Fish spinal cord	[15]
		Mouse forebrain	[16,17]
		Mouse mid-/hindbrain	[17]
(3) Vessel maturation/BBB formation	WNT7A/7B	Mouse spinal cord	[18,19]
(4) Vascular stability (via TGFβ activation)	Integrins αvβ6 and αvβ8	Mouse forebrain	[20,21]

#### Vascular endothelial growth factor (VEGF)

The VEGF family of secreted glycoproteins is the best-studied group of angiogenic molecules to date. The importance of VEGF-A for mammalian development is demonstrated by the early lethality of mouse embryos that lack even one copy of the *Vegfa* gene that encodes VEGF-A [33]. In these mice, ECs do not differentiate, and blood vessels accordingly cannot assemble. This phenotype is also observed in mice lacking both copies of the *Kdr* gene, which encodes the VEGF-A receptor tyrosine kinase VEGFR2, also known as FLK1 or KDR [34]. VEGF-A binding to VEGFR2 also activates intracellular signalling cascades in ECs that drive their proliferation for vascular expansion [35,36]. Furthermore, VEGF-A acts as a guidance cue for sprouting vessels by directing the filopodia that extend from the endothelial ‘tip cells’ to lead the vessel sprout into avascular areas [37,38].

The *Vegfa* mRNA transcripts include several differentially spliced forms that are translated into VEGF-A isoforms of varying lengths, which possess varying affinities for the extracellular matrix (ECM) and different receptor binding properties [39]. In the mouse, the main isoforms are termed VEGF120, VEGF164 and VEGF188. The corresponding human isoforms are all one amino acid longer in length and accordingly termed VEGF121, VEGF165 and VEGF189, respectively. VEGF120 is a diffusible isoform with low ECM affinity that binds VEGFR2, whilst the VEGF164 and VEGF188 isoforms have a high ECM affinity and bind both VEGFR2 and the non-catalytic receptor neuropilin 1 (NRP1) [40] (see below). All VEGF isoforms promote EC proliferation similarly, and therefore each type is sufficient for vessel formation during embryonic development [38]. However, the differential affinity of the isoforms for the ECM is essential for proper vascular patterning in the developing brain. Accordingly, mouse embryos expressing only VEGF120 exhibit defective branching of vessels in the hindbrain because VEGF-A fails to form proper growth factor gradients to guide the tip cell filopodia [38]. As the ECs in the growing vessels continue to proliferate, the vessels in mice expressing VEGF120 only increase their luminal diameter excessively. Vice versa, the vessels of mice expressing only VEGF188 as the isoform that is most tightly bound to ECM show vascular hypersprouting, leading to excessively thin vessels [38]. Together, these findings show that the different VEGF-A isoforms are cooperatively required for proper vascular morphogenesis in the brain [38].

VEGF164 and VEGF188 can also bind to a co-receptor complex composed of VEGFR2 and NRP1, with the VEGF isoforms believed to form a bridge between both receptors [41]. However, VEGF-A does not require NRP1 to direct angiogenesis in the developing brain; this is illustrated by the finding that both forebrain and hindbrain vascularisation are unaffected in mouse embryos that express NRP1 with a defective binding pocket for VEGF-A [42,43]. Nevertheless, embryo-wide or endothelial *Nrp1* deletion impairs CNS vascularisation [5,6,44], suggesting that NRP1 acts in ECs to promote brain vascularisation through a VEGF-independent signalling mechanism. This mechanism may involve the modulation of TGF-β signalling [45,46] and the promotion of tip cell filopodia extension and actin cytoskeletal reorganisation via integrin-associated signalling pathways [47].

Experiments in avian embryos support the idea that NPCs modulate the ingression of PNP vessels into the neural tube by releasing VEGF-A [8]. *Vegfa* is expressed abundantly in the spinal cord at a stage when it is composed mainly of NPCs [8]. Furthermore, ectopic expression of VEGF165 and VEGF189 in the neural tube, achieved through targeted *in ovo* electroporation of expression vectors, induces supernumerary radial vessel entry points into the neural parenchyma [11].

NPC expression of VEGF-A is also required for neural tube vascularisation in the zebrafish [15], with fish VEGF-A encoded by not one, but two genes that are termed *vegfaa* and *vegfab* [48]. *Vegfaa* and *vegfab* are collectively expressed across the neuroepithelium, including in NPCs and the neuron-populated mantle zone [48]. The depletion of *vegfab* from radial glia, the predominant NPC subtype in the fish spinal cord, prevented the formation of the bilateral vertebral arteries that flank the neural tube and represent the fish equivalent of the PNP [15]. Preventing their formation subsequently blocks the vascularisation of the zebrafish neural tube from these vessels [15]. Inhibiting endothelial responsiveness to both VEGF-A ligands by mutating *kdrl*, the fish orthologue of mammalian VEGFR2, also blocks the formation of the basilar artery and sprouting of the central arteries in the brain [48].

**Figure 2:**
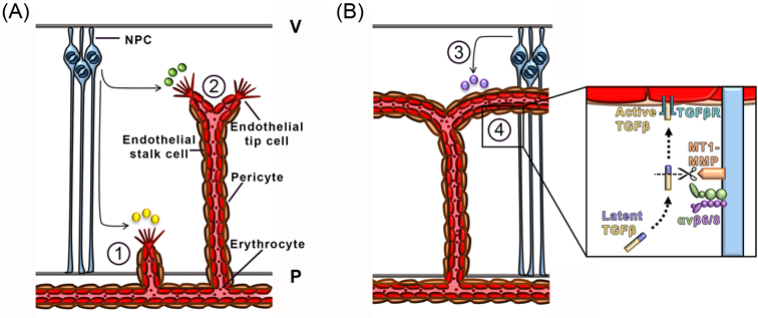
NPC regulation of CNS vascularisation in the developing brain Schematic representation of molecular mechanisms by which NPCs promote CNS vascularisation. (**A**) NPC-derived secreted cues such as VEGF and WNT ligands promote vascular ingression and radial outgrowth, indicated with (1), and lateral branching, indicated with (2), respectively. (**B**) NPC-secreted WNT ligands and NPC-activated TGFβ regulate vascular maturation and stability, respectively, indicated with (3) and (4). Inset, NPCs (light blue) express integrins αvβ6 or αvβ8 (green/purple) to promote the cleavage of latent TGFβ (blue/yellow) by the matrix metalloprotease MT1-MMP (orange) to release active TGFβ (yellow). Active TGFβ then binds TGFβ receptors (cyan) on ECs (red). Abbreviations: P, pial surface; V, ventricular surface.

NPCs have been shown to be the main cellular source of VEGF-A during mammalian CNS vascularisation. Thus, loss of *Vegfa* expression from NPCs perturbs cortical vascular development in mice [16,17]. Specifically, both the number of endothelial filopodia and filopodial length, and consequently vessel branching and vascular coverage, are decreased in the cortex of mouse embryos with reduced, but not absent *Vegfa* expression, demonstrating that brain angiogenesis is regulated by VEGF-A in a dose-dependent manner [16] (Figure 2A). These experiments were performed by Cre-LoxP-mediated recombination of one conditional *Vegfa* null allele in cells expressing the NPC gene nestin, together with a hypomorphic mutation of the second *Vegfa* allele [16]. This strategy was required to overcome the effect of nestin-driven, undesired germline expression of *Cre*, which effectively rendered the *Vegfa* deletion embryo-wide in the F1 generation [33,16]. Thus, a male carrying the *Nestin-Cre* transgene and a hypomorphic *Vegfa* allele was mated to females carrying conditional *Vegfa* null alleles to obtain embryos with an approximately 75% reduction in brain VEGF-A levels. The consequence of complete loss of *Vegfa* expression from NPCs on brain vascularisation has therefore not yet been demonstrated.

VEGF-A signalling in the CNS is modulated positively by the hypoxia-inducible factor 1α (HIF1A). HIF1A is up-regulated and stabilised in cells under low oxygen tension to increase VEGF-A expression, which then helps to attract new blood vessels into the hypoxic areas to restore normoxia e.g. in the tumour microenvironment [49]. HIF1A is expressed by NPCs prior to the onset of brain vascularisation [50,51] and is thought to regulate VEGF-A expression to drive angiogenesis into avascular regions of the expanding neuroepithelium [51]. Thus, mouse embryos lacking HIF1A specifically in NPCs following Cre-LoxP mediated recombination of conditional null *Hif1a* alleles, under the control of the nestin promoter, have reduced tissue-wide VEGF-A levels and reduced blood vessel extension into the cortical plate [51]. Furthermore, cortical vasculature regresses at later stages of gestation in these mutants, indicating that HIF1A-driven VEGF expression supports vascular maintenance [51].

VEGF-A signalling in the CNS is modulated negatively by a VEGF-A binding decoy receptor termed soluble (s) fms-related tyrosine kinase 1 (FLT1). Thus, sFLT1 expression in the zebrafish neural tube, regulated non-cell autonomously by resident radial glia, restricts VEGF-induced radial vessel ingression into the spinal cord to specific sites and limits oversprouting within the parenchyma, thus demonstrating that endogenous *flt1* expression is essential for normal CNS vascularisation [11,52]. It is not yet known whether vertebrate NPCs modulate vessel ingression into the neural tube through secreting sFLT1. Nevertheless, post-mitotic MNs have been shown to express sFLT1 in the chick to delay VEGF-induced vascularisation of their own domain, the nascent bilateral motor columns in the ventral spinal cord [13]. Specifically, it was shown that blocking *Flt1* expression in the MNs of the avian spinal cord via forced expression of *Flt1*-targeting microRNAs under a MN-specific promoter caused ectopic vessel ingression sites and precocious motor column vascularisation [13]. Studies in fish demonstrate also that neuronal *flt1* prevents ectopic vessel sprouting in the neural tube, and its function is therefore conserved across different vertebrate species [53].

#### WNTs

The WNT family of secreted ligands is crucial to many developmental mechanisms, including brain vascular development [54]. WNT proteins bind to a co-receptor complex comprised of a lipoprotein receptor-related protein (LRP) and a frizzled (Fz) receptor to inhibit the proteolytic degradation of β-catenin and therefore the translocation of β-catenin to the nucleus, where it would otherwise act as a transcriptional coactivator. Thus, β-catenin forms part of the TCF/LEF transcription factor complex that drives the expression of genes responsible for processes such as cell cycle progression and fate determination.

WNT proteins can influence EC behaviour *in vitro*; for example, WNT7A promotes the migration of cultured ECs across a fibronectin-coated filter, suggesting that it acts as a guidance cue [19]. WNT7A and WNT7B are expressed in the murine CNS, and the analysis of genetic mouse mutants lacking these WNT ligands has highlighted their important roles in establishing and maintaining CNS vascularisation [18]. Specifically, the combinatorial deletion of conditional null alleles of the *Wnt7a* and *Wnt7b* genes in NPCs with *Cre* expressed under the control of either the *Sox2* or *nestin* promoters shows that WNT signalling is required for vascularisation of the mouse CNS [18] (Figure 2A). Vascular outgrowth is impaired in the spinal cord of *Wnt7a/7b* compound mutants; however, ablation of either ligand alone has no effect on neural tube vascularisation, demonstrating that they can compensate for each other during this process [18]. A large number of other WNTs are also expressed across the forebrain and spinal cord [19], but it is not yet known if they too are required for CNS vascularisation, or instead drive other aspects of CNS development, such as neuronal specification [55].

WNT signalling is also necessary for establishing patent and selectively permeable CNS vasculature (Figure 2B). Firstly, NPC-specific *Wnt7a/b* mutant embryos have haemorrhagic brain vasculature, in addition to the angiogenesis defects described above [18]. Secondly, *Wnt3a*, expressed in mouse forebrain and spinal cord [19], promotes the expression of the tight junction protein claudin 3 in cultured brain microvascular ECs [56]. Thirdly, the conditional inactivation of WNT signalling in brain ECs through endothelial deletion of β-catenin increases the extravasation of injectable vascular permeability tracers into the brain [56,57]. Finally, NPC-derived WNT signals promote GLUT1 expression in ECs and therefore enable glucose transport into the brain, further demonstrating the importance of WNT proteins in establishing the BBB [18].

Despite being important for blood vessel formation, another study proposed that prolonged WNT signalling induces vascular regression and prevents vessel stabilisation during embryogenesis and thereby causes vascular leakage in the cortex after birth [58]. In particular, radial glia loss in the developing forebrain by conditional *Orc3* deletion results in ectopic activation of WNT signalling in the SVP at later stages of gestation; this consequently impairs vessel maturation through increased expression of matrix metalloproteases that mediate vascular remodelling [58]. In agreement, vessel regression is not as severe in mouse mutants with radial glia ablation on a *Wnt7b*-null background, or after pharmacological blockade of WNT signalling or inhibition of metalloprotease function [58]. It seems possible that NPCs regulate WNT signalling in ECs through other molecules, such as through NOTCH-based regulation of β-catenin [59].

Recent research suggests that the orphan G-protein coupled receptor, GPR124, plays a role in WNT-regulated angiogenesis and BBB formation. Thus, *Gpr124^−/−^* mutant mice possess cerebrovascular malformations in different areas of the developing CNS that are characteristic of defective WNT signalling, including poor endothelial barrier maturation and haemorrhaging [60–64]. However, it is not yet known which ligand (if any) stimulates GPR124 and whether this signalling pathway operates in NPCs.

#### Integrins

Integrins are heterodimeric membrane-bound receptors that are composed of an α and a β subunit and are vital for establishing tissue architecture and maintaining structural integrity during development. Integrins regulate cell behaviour by transducing signals after binding cell contact-dependent cues in the ECM, such as fibronectin and laminin. NPCs express integrins abundantly during neural development to transduce signals from the basal lamina into NPCs, and also between NPCs, to dictate the onset of neurogenesis (e.g. [65,66]).

In the developing CNS, integrins also promote normal vascular development by modulating transforming growth factor beta (TGF-β) signalling (Figure 2B). In particular, both αvβ6 and αvβ8 complexes promote cleavage of pro-TGF-β by the matrix metalloprotease MT1-MMP to convert TGF-β into an active form that is able to signal to ECs [67–69]. As TGF-β promotes vessel stabilisation and BBB establishment [70,71], mice lacking the integrin subunits αv or β8 have brain vascular defects reminiscent of TGF-β mutants [20,21]. Specifically, the Cre-LoxP-mediated ablation of the *Itgb8* gene that encodes β8 in *nestin*-expressing NPCs disrupts vessel morphology, in addition to compromising the adhesion of NPCs to each other [20]. In contrast, ablating β8 integrin later on in cortical neurons does not impair vascularisation [20]. The NPC-specific deletion of the αv integrin subunit also results in abnormal cerebral vascularisation and brain haemorrhage [21]. These findings, coupled with the knowledge of integrin-based activation of latent TGF-β, are consistent with a role for NPCs in helping to stabilise nascent vessels by modulating endothelial TGF-β signalling [20,21]. Interestingly, mice lacking either αv or β8 expression in NPCs reach birth and do not exhibit vascular leakage postnatally, suggesting that unidentified mechanisms can compensate for their function during postnatal CNS vascularisation [20,21].

#### Netrins

Netrins are laminin-related secreted molecules that bind to UNC5 and DCC receptors to exert repulsive or attractive actions in axons, respectively. Netrin signalling can also have pro- or anti-angiogenic effects during developmental angiogenesis, but it is not known whether this is due to the use of alternative receptors [72]. It has also been discussed that differing netrin roles may be explained by different netrin concentrations or the initiation of alternate cellular behaviours depending on the nature of the recipient cell [72]. For example, one study reported that netrin promotes EC survival by binding to and subsequently preventing pro-apoptotic signalling through UNC5B in ECs [73], whilst another found that netrins promote post-ischaemic neovascularisation [74]. Yet another study found that UNC5B is required to restrict vessel branching and ectopic vessel growth in the CNS [75]. In particular, blood vessels in the *Unc5b*-null mouse cortex and spinal cord and the morpholino-treated zebrafish neural tube have significantly more tip cell filopodia [75]. However, mice lacking netrin 1 do not possess any obvious defects in CNS vascularisation, suggesting that other netrins can compensate for netrin 1 in this process [75]. In support of this idea, both netrins 1 and 4 are expressed abundantly in the mouse forebrain and spinal cord [76,77]. As they are expressed highly in the ventricular zone, they may be secreted by NPCs to restrict vessel growth in the germinal region during neurogenesis. However, NPC-specific compound *netrin* mutants have not been analysed to date to investigate these possibilities, and additional work is therefore required to understand the complexity of mechanisms by which netrin signalling affects ECs.

## Role of vascular signals in NPC regulation and neurogenesis

During neurogenesis in vertebrate embryos, NPCs expand in number and specialise to give rise to partially committed progenitors that then differentiate into diverse subtypes of neurons and glia. Initially, the neuroepithelium contains primitive NPCs [2], which differentiate into a self-renewing NPC subtype called apical radial glia. Both subtypes make up the broader class of NPCs known as ‘apical progenitors’ (APs) that are defined primarily by their position in the neuroepithelium during mitosis and reside in the apical portion of the neurogenic zone [78,79]. At the beginning of CNS development, APs divide rapidly to expand in number and establish a large progenitor pool. Following expansion, APs differentiate into ‘basal progenitors’ (BPs), a subclass of NPCs that contains a varying proportion of basal radial glia and resides in the basal portion of the neurogenic zone [80]. Basal radial glia are sparse in the forebrain of lower mammals with smooth (lissencephalic) brains such as rodents, but are more prevalent in higher mammals with larger and gyrified (gyrencephalic) brains, including humans [81]. APs and BPs are the most abundant types of progenitors in the early mammalian brain, but other progenitor subtypes with intermediate properties are also present in smaller numbers [82].

The symmetry of NPC cell division controls whether progenitors self-renew or differentiate into more committed NPCs and post-mitotic neurons [83]. The division mode is linked closely to cell cycle progression and the polarised distribution of intracellular fate determinants (e.g. [84,85]). These two processes are regulated by molecular cues originating from the surrounding tissue, which therefore acts as a ‘regulatory niche’. Many of these extrinsic signals are generated by other neural cells [86] and also by vasculature, as discussed below (Table 2).

**Table 2:** Vascular regulation of Neurogenesis

Vessel-derived signals	NPC target	Murine CNS region	Effect	References
Oxygen	HIF1A	Forebrain	AP differentiation into BPs	[22]
Secreted signals, unidentified	Unidentified	Forebrain-derived neurospheres hindbrain	NPC self-renewal	[23,24]
Extracellular matrix, unidentified component(s)	ITGB1	Ventral forebrain	Vessel anchorage/proliferation of APs for interneuron generation	[25]

### Blood vessels regulate NPC commitment along specific lineages through tissue oxygenation

**Figure 3:**
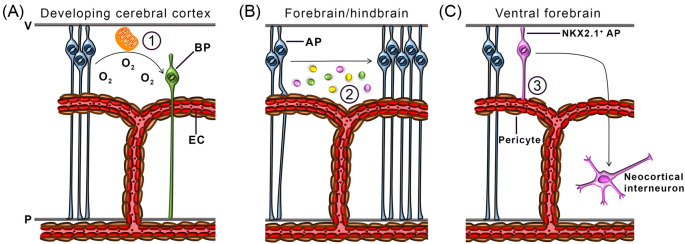
Vascular regulation of embryonic NPCs Schematic representation of molecular mechanisms by which blood vessels regulate NPC behaviour in the embryonic brain. (**A**) APs (blue) in the developing cerebral cortex differentiate into BPs (green) following a switch in metabolism, which is enabled by blood vessel-derived oxygen. (**B**) ECs secrete unidentified diffusible factors (indicated by pink, green and yellow spheres), which promote AP self-renewal and expansion in the forebrain and hindbrain. (**C**) NKX2.1^+^ APs in the ventral forebrain attach to unidentified matrix components on periventricular vasculature via an integrin containing the β1 subunit. Vessel anchorage maintains NKX2.1^+^ AP proliferation and therefore enhances their generation of neocortical interneurons. Abbreviations: P, pial surface; V, ventricular surface.

Two studies have shown that blood vessels promote NPC differentiation into more committed subtypes. Firstly, ectopic *Vegfa* expression in the mouse cortex, achieved through *in utero* electroporation, promotes angiogenesis in the targeted areas and is accompanied by the supernumerary generation of TBR2^+^ BPs [87]. Newly-formed BPs are generated in regions far from the ventricle, but close to ectopically sprouting blood vessels, suggesting that vasculature facilitates the conversion to, or the expansion of, this NPC subclass [87]. Even though it was not tested whether the excessive generation of BPs occurred at the expense of less differentiated progenitors or if it represents a selective amplification of BPs, this study suggested that VEGF-induced blood vessels alter NPC commitment along specific lineages. In agreement with this idea, a recent study showed that blood vessels regulate NPC commitment by alleviating tissue hypoxia and altering NPC metabolism [22] (Figure 3A). Firstly, the onset of cortical vascularisation relieves local tissue hypoxia, which in turn destabilises HIF1A [22]. Secondly, it was shown that these processes correlate temporally with a decline of PAX6^+^ APs and an increase of TBR2^+^ BPs, suggesting increased progenitor commitment [22]. Thirdly, preventing cortical vascularisation through endothelial GPR124 deletion (discussed above) increased hypoxia and therefore HIF1A levels, which resulted in NPC expansion at the expense of commitment to a BP fate [22]. Finally, HIF1A was shown to control NPC fate by maintaining NPC metabolism in a state of glycolysis, favoured by APs, and concomitantly preserving expression of the stemness gene *Myc* [22].

### Endothelial cells secrete factors that prevent NPC differentiation

The first indication of angiocrine signalling to NPCs was demonstrated when NPCs were propagated as ‘neurospheres’ derived from e10 mouse cortical tissue and co-cultured with either bovine pulmonary artery or adult brain microvascular EC lines [23]. By using a transwell system that physically separated both cell types, it was shown that ECs released secreted factors that stimulated neurosphere expansion significantly more than co-culture with non-ECs [23]. The EC-conditioned media promoted a stem-like character in the cultured NPCs, because they formed neurospheres comprised of many more NPCs than neurons [23]. In fact, single NPCs propagated in co-culture with ECs did not produce any neurons during a 4-day time-lapse video recording, indicating that soluble factors produced by ECs promote NPC self-renewal and delay neurogenesis, at least *in vitro* [23]. More recently, studies of the mouse embryonic hindbrain have suggested that neural vasculature regulates NPC self-renewal by releasing diffusible regulatory signals also *in vivo* (Figure 3B). Hindbrain progenitors divide most actively following the main phase of vascularisation, and the temporal profile of progenitor mitosis is disturbed by impairing vessel outgrowth in the NPC-populated germinal zone [24]. Thus, constitutive and endothelial NRP1 mutant hindbrains are poorly vascularised at early phases of hindbrain neurogenesis [5,24], which results in failed cell cycle re-entry of early-formed NPCs and their increased differentiation into neural cell types [24]. Taken together, these findings illustrate that the embryonic endothelium supports NPC expansion, for example by secreting regulatory molecules.

### Molecular regulation of neurogenesis by vascular signals

#### VEGF regulation of NPCs

As discussed above, several lines of evidence suggest that NPCs secrete VEGF-A to promote CNS vascularisation. Vice versa, blood vessels have been suggested to also release VEGF-A to regulate neurogenesis. In one study, conditional mouse mutants lacking *Vegfa* expression in *Tie2*-expressing ECs were reported to have profound cortical malformations and heterotopias [88]. Yet, the precise mechanisms involved have not yet been elucidated. On the one hand, it was not examined whether a specific neural VEGF-A receptor might be required for this process; on the other hand, it was reported that endothelial *Vegfa* deletion disrupts forebrain vascularisation [88] and vascular homoeostasis [89], and an endothelial *Vegfa* mutation may therefore affect cortical development indirectly by compromising the vasculature, rather than by impairing direct VEGF-A signalling to NPCs or their progeny.

Another study sought to identify whether VEGF-A signals directly to embryonic NPCs. Recombinant VEGF165 was found to promote the formation of neurospheres *in vitro* after serial passaging, but this effect was lost in VEGFR2-deficient neurospheres [90]. In contrast, other observations, made in the developing mammalian CNS, argue against direct effects of VEGF-A on NPCs. Firstly, two independent studies show that murine NPCs lack VEGFR2 expression *in vivo*, with one study having examined the embryonic mouse forebrain mid-way through cortical neurogenesis [87], and the other having studied the embryonic mouse hindbrain through the main phases of hindbrain neurogenesis [24]. Secondly, the alternative VEGF-A receptor NRP1 is expressed in hindbrain NPCs, but their proliferation is unaffected by progenitor-specific NRP1 ablation [24]. The divergent results obtained for VEGFR2 roles in NPCs through culture models versus *in vivo* studies may be that NPCs up-regulate VEGFR2 after they are cultured. It is not known why this might occur, but one possible explanation could be that dissociation of NPCs from other cells within the neuroepithelium alters NPC gene expression patterns.

#### Blood vessels regulate neurogenesis via cell contact with NPCs

Many stem cell populations share physical contacts with neighbouring cells, from which they receive paracrine niche signals. For example, adult neural stem cells (NSCs) project endfeet onto periventricular blood vessels and receive endothelial-derived notch and ephrin signals to remain in quiescence [91,92]. In analogy, a fate-restricted subset of forebrain NPCs contacts the SVP in the ventral telencephalon [25]. Accordingly, it was shown that NKX2.1^+^ radial glial cells in the medial ganglionic eminence, which are destined to generate neocortical interneurons, project endfeet onto periventricular ECs in pericyte-free vessel areas [25]. This adhesion is, at least in part, integrin-mediated, because genetic targeting of the *Itgb1* gene causes process retraction. This detachment subsequently impairs the mitotic capacity of the progenitors, as well as their ability to generate interneurons [25] (Figure 3C). In agreement, the disruption of vessel networks by injecting function blocking antibodies against VEGFR2 into the forebrain ventricle also disrupts anchorage of these NPCs to blood vessels and reduces their mitotic capacity [25]. However, the specific integrin ligands involved in radial glia anchorage to blood vessels for neurogenic regulation remain unidentified. Notably, very few radial glia in the dorsal telencephalon terminate endfeet on cortical blood vessels [25]. Vessel association therefore appears to be important for specific NPC subsets.

## Conclusions

The studies described above demonstrate the importance of extensive molecular crosstalk between NPCs and ECs in the developing CNS of several vertebrate species. We have discussed how NPCs direct vascularisation of the neuraxis through a number of complementary signalling mechanisms that regulate the initial invasion of the parenchyma by blood vessels, as well as their branching and eventual maturation within the neural tube. Yet, many outstanding questions remain to be answered before we will fully understand the process of CNS vascularisation. For example, we know now that VEGF-A has a key role in promoting mammalian CNS vascularisation, but we still need to define how it cooperates with other signalling pathways discussed here or described elsewhere to fine-tune the formation and maturation of neural vasculature. Moreover, it remains to be investigated which of these pro-angiogenic factors are of NPC or neuronal origin. Answering these questions may help devise effective therapies aimed at regenerating defective or regressed cerebrovasculature in ischaemic or degenerative CNS diseases.

We have also discussed recent studies that highlight the importance of blood vessels in regulating neurogenesis. However, the specific mechanisms involved appear to differ, at least in part, across different regions of the developing CNS. For example, we have described above that a subset of forebrain NPC processes terminate with endfeet on periventricular vessels [22], whereas hindbrain NPC processes are more loosely associated with germinal zone vasculature [24]. Moreover, we have discussed that restoring normoxia in poorly vascularised forebrains rescues hypoxia-induced NPC commitment defects in the forebrain [22], even though restoring normoxia does not prevent precocious NPC commitment in the hindbrain of mouse mutants possessing an avascular hindbrain germinal zone [24]. These regional differences in NPC behaviour may stem from differences in the NPC subtypes present in each brain region; for example, hindbrains lack basal TBR2^+^ NPC subtypes [93], whose generation in the forebrain depends on vascularisation-driven relief from hypoxia [22]. Indeed, prior studies having focused predominantly on neurogenesis regulation within the context of only one defined subregion of the developing brain. Future work should therefore compare different parts of the embryonic CNS in a systematic manner to distinguish the cellular and molecular mechanisms that are shared between different brain regions from those that are unique to specific compartments.

Extending the idea that blood vessels regulate embryonic neurogenesis via oxygen provision, both forebrain and hindbrain studies have shown that blood vessels additionally provide angiocrine signals that regulate NPC behaviour, independently of oxygenation [23,24]. This observation is reminiscent of findings in the adult neurogenic niche, where ECs in the lateral subventricular zones regulate resident NSC behaviour [94]. However, whether the angiocrine signals in the adult and embryonic niches are similar or distinct remains to be established. In particular, it will be interesting to examine whether embryonic NPCs respond to contact-dependent vascular regulation via notch-delta signalling [91] or endothelial cytokines such as PEDF, PLGF, BDNF or NTF3 [94–97], which are all key for stem and progenitor cell regulation in the adult neurogenic niche. This is an important consideration, because findings made in the adult brain are not necessarily predictive of angiocrine regulation of NPCs *in utero*, as demonstrated, for example, by the divergent roles of NTF3 during adult and embryonic neurogenesis [97,98]. It will also be intriguing to define whether ECs in extra-neural vessel networks contribute to the neurogenic niche, in particular in the case of the PNP, which is close to NPC endfeet contacting the basal surface of the neuroepithelium [24] and is therefore ideally placed to provide diffusible regulatory cues.

Ultimately, the knowledge gained on the vascular regulation of embryonic neurogenesis will increase our understanding of CNS formation and hopefully lay the foundation for innovative repair strategies in adult brain disease by exploiting developmental principles. For example, activating vasculature in the adult brain may be an ideal means to stimulate existing NSC niches and also create new niches that can support the survival and function of transplanted neural stem and progenitor cells for adult brain repair [99–102].

## Abbreviations

AP: apical progenitor
BBB: blood–brain barrier
BP: basal progenitor
CNS: central nervous system
EC: endothelial cell
ECM: extracellular matrix
FLT1: fms-related tyrosine kinase 1
HIF1A: hypoxia-inducible factor 1α
MN: motor neuron
MT1-MMP: membrane type 1 metalloprotease
NKX2.1: NK2 homeobox 1
NPC: neural progenitor cell
NRP1: neuropilin 1
NSC: neural stem cell
PAX6: paired box protein Pax-6
PNP: perineural vascular plexus
SVP: subventricular vascular plexus
TBR2: T-box brain protein 2
TGF-β: transforming growth factor beta
VEGF: vascular endothelial growth factor
VEGFR2: vascular endothelial growth factor receptor 2
